# Detailed Transcriptome Description of the Neglected Cestode *Taenia multiceps*

**DOI:** 10.1371/journal.pone.0045830

**Published:** 2012-09-25

**Authors:** Xuhang Wu, Yan Fu, Deying Yang, Runhui Zhang, Wanpeng Zheng, Huaming Nie, Yue Xie, Ning Yan, Guiying Hao, Xiaobin Gu, Shuxian Wang, Xuerong Peng, Guangyou Yang

**Affiliations:** 1 Department of Parasitology, College of Veterinary Medicine, Sichuan Agricultural University, Ya’an, China; 2 Department of Chemistry, College of Life and Basic Science, Sichuan Agricultural University, Ya’an, China; Queensland Institute of Medical Research, Australia

## Abstract

**Background:**

The larval stage of *Taenia multiceps*, a global cestode, encysts in the central nervous system (CNS) of sheep and other livestock. This frequently leads to their death and huge socioeconomic losses, especially in developing countries. This parasite can also cause zoonotic infections in humans, but has been largely neglected due to a lack of diagnostic techniques and studies. Recent developments in next-generation sequencing provide an opportunity to explore the transcriptome of *T. multiceps*.

**Methodology/Principal Findings:**

We obtained a total of 31,282 unigenes (mean length 920 bp) using Illumina paired-end sequencing technology and a new Trinity de novo assembler without a referenced genome. Individual transcription molecules were determined by sequence-based annotations and/or domain-based annotations against public databases (Nr, UniprotKB/Swiss-Prot, COG, KEGG, UniProtKB/TrEMBL, InterPro and Pfam). We identified 26,110 (83.47%) unigenes and inferred 20,896 (66.8%) coding sequences (CDS). Further comparative transcripts analysis with other cestodes (*Taenia pisiformis*, *Taenia solium*, *Echincoccus granulosus* and *Echincoccus multilocularis*) and intestinal parasites (*Trichinella spiralis*, *Ancylostoma caninum* and *Ascaris suum*) showed that 5,100 common genes were shared among three *Taenia* tapeworms, 261 conserved genes were detected among five Taeniidae cestodes, and 109 common genes were found in four zoonotic intestinal parasites. Some of the common genes were genes required for parasite survival, involved in parasite-host interactions. In addition, we amplified two full-length CDS of unigenes from the common genes using RT-PCR.

**Conclusions/Significance:**

This study provides an extensive transcriptome of the adult stage of *T. multiceps*, and demonstrates that comparative transcriptomic investigations deserve to be further studied. This transcriptome dataset forms a substantial public information platform to achieve a fundamental understanding of the biology of *T. multiceps*, and helps in the identification of drug targets and parasite-host interaction studies.

## Introduction

*Taenia multiceps* is a taeniid cestode, which inhabits the small intestine of dogs and other canids (foxes, wolves, and jackals), making these definitive hosts a widespread infection reservoir [1]. The coenurus (larva of *T. multiceps*) parasitizes the central nervous system (CNS) of sheep, occasionally goats, deer, antelopes, chamois, rabbits, hares and horses, and less commonly, cattle [2]–[5]. It frequently causes the death of infected animals, and can lead to huge economic losses of sheep/goats, predominantly in developing countries, such as those in Africa and southeastern Asia [6]. The parasite can also cause zoonotic infections in humans, leading to serious pathological conditions in humans, which occur more commonly than previously assumed [6]–[11].

The gravid proglottids of *T. multiceps* are discharged from infected dogs and are ingested by intermediate hosts (including humans, especially in rural grazing areas where people raise sheep or other ungulates, and keep guard dogs in close proximity) through contaminated food or water [12]. The proglottids then release oncospheres in the intestine and penetrate the intestinal mucosa and blood vessels. After reaching the brain through the bloodstream, they will take 2–3 months to grow into a coenurus causing increased intracranial pressure. This will lead to the onset of clinical signs, such as ataxia, hypermetria, blindness, head deviation, headache, stumbling and paralysis [13]–[15]. Once the tissue of infected sheep or other livestock has been ingested by a definitive host, the lifecycle is completed, and the parasites develop into adult tapeworms in the small intestine of the host [5].

Together with this complex lifecycle, the specific immune evasion traits of parasites and even the randomness of the infection make research and drug or vaccine programs for *Taenia* species very difficult; consequently, new methods to control this parasite are required. Although traditional methods of control (such as burning out the infected brain and spinal cord of sheep, or deworming infected dogs with anti-parasitic drugs) help to disrupt the lifecycle of this parasite, its global distribution still includes Europe [16]–[19], North America [8], [9], Africa and Asia [6], [20].

Despite its global importance, available gene sequences for *T. multiceps* remain scarce. Currently, only 101 nucleotide sequences and 103 proteins have been published on NCBI and only the mitochondrial genome of *T. multiceps* has been sequenced [21]. To improve the control of this parasitic cestode, the identification of molecular targets to develop new effective anti-parasitic drugs are necessary. Recent developments in next generation sequencing (NGS) technologies [22]–[25] and recent progress in bioinformatics, such as the new Trinity de novo assembling program [26], make it possible to explore the fundamental biology of cestodes in far greater detail than currently available information, and are cheaper than the commonly used Sanger sequencing technique [27]. To date, a fractionated transcriptome of the human parasite, *Taenia solium* cysticerca, has been revealed by the ORESTES method [28], and a cDNA library has been constructed [29] with 30,700 ESTs available in GenBank. Some vaccine and diagnostic targets were proposed from this study. However, further datasets generated by high-throughput sequencing and comparative transcriptome analysis could bring a more comprehensive understanding of parasite biology. To our knowledge, the transcriptomes of *Taenia pisiformis*, *T. solium*, *Echinococcus granulosus*, *Echinococcus multilocularis* and *Hymenolepis microstoma* by NGS technology have already been studied, but only *T. pisiformis* transcriptome dataset has been published [30], [31]. Compared with nematodes [32]–[38] and trematodes [39]–[42], published transcriptome data of cestodes by NGS remain scarce. An improved understanding of the entire molecular transcriptome of the adult stage of this cestode is necessary. This can provide a platform to execute the identification or validation of required genes and gene products in the design of cestocides aimed at controlling the infection in dogs and disrupting the lifecycle of *T. multiceps* [19], [32].

Here, we used the novel assembler Trinity and Illumina sequencing technology to gather initial insights into the transcriptome of the adult stage of *T. multiceps*. In addition, a comparative analysis was performed against the transcriptomes of other cestodes, including *T. pisiformis*, *T. solium*, *E. granulosus* and *E. multilocularis*, and intestinal parasites, including *T. spiralis*, *A. caninum* and *A. suum*, in order to help us to discover essential biological pathways and pathway-related genes for intestinal parasites or cestodes specifically. These would be essential for parasite development and survival, or for parasite-host interactions [33], [34], [43]. Therefore, we anticipate that a better understanding of parasite development or parasite-host interaction at a molecular level may help to develop new anti-cestode drug targets or candidate vaccines [35].

## Results

### Illumina Sequencing and Assembly

**Figure 1:**
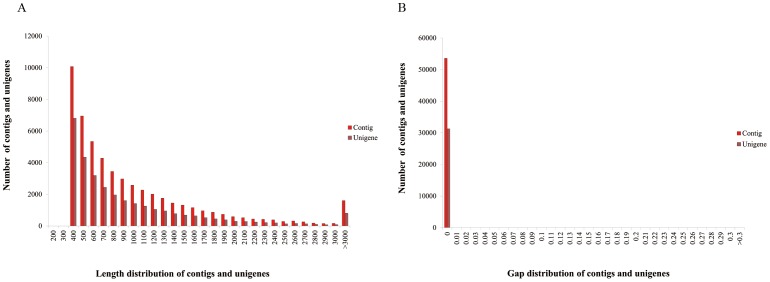
Overview of the *T. multiceps* transcriptome by Trinity assembling.

In order to generate a broad survey of genes involved in *T. multiceps* survival and development, the RNA of *T. multiceps* adults was extracted. Using Illumina (paired-end) sequencing technology, we obtained a total of 28.3 million raw reads with an average length of 90 bp, a total of approximately 2.55 Gigabase pairs (Gbp). After the removal of raw reads that only had 3' adaptor fragments, ambiguous reads and low-quality reads, 27.4 million (2.47 Gbp, 96.92% of the raw reads) clean reads with a Q20 percentage of 89.92% and a GC content of 49.04% remained. All clean reads were assembled de novo by Trinity, generating 53,568 contigs without gaps longer than 300 bp (5,218 Mbp), with a mean contig length of 974 bp and a N50 of 1,268 bp (Table 1). A total of 36,538 contigs (68.21%) were longer than 500 bp. To join further sequences and remove any redundant sequences, contigs were clustered using the TIGR Gene Indices clustering tools (TGICL). A total of 31,282 unigenes were produced by the clustering, with an average length of 920 bp and a N50 of 1,206 bp (Table 1). Of these, 20,123 unigenes (64.33%) were longer than 500 bp, and 9,504 unigenes (30.38%) were longer than 1,000 bp. Short contigs and unigenes (less than 300 bp) were removed, the maximum length of both contigs and unigenes was 11,875 bp (Table 1). The length distribution of these contigs and unigenes is shown in Figure 1A. The gap distribution of contigs and unigenes was analyzed to identify the data quality. All contigs and unigenes showed no gaps, thus demonstrating the high quality of Trinity assembling (Figure 1B).

**Table 1:** Transcriptome summary of the adult stage of *T. multiceps* and detailed bioinformatics annotations.

**Raw sequences and Assembly statistics**	
Raw reads	28,320,027
Clean reads	27,447,770, each 90 bp in length
GC content	49.04%
Contigs (≥300) (mean length; max; N50)	53,568 (974 bp; 11,875 bp; 1,268 bp)
Unigenes (≥300) (mean length; max; N50)	31,282 (920 bp; 11,875 bp; 1,206 bp)
**Bioinformatics annotations of ** ***Tm*** ** unigenes**	
Gene annotation against animal proteins of Nr	17,618 (56.3%)
Gene annotation against Drosophila protein of Nr	5,925 (18.9%)
Gene annotation against UniProtKB/Swiss-Prot	14,350 (45.9%)
Gene annotation against UniProtKB/TrEMBL	16,286 (52.1%)
Gene annotation against COG	6,653 (21.3%), 24 categories
Gene annotation against KEGG	11,645 (37.3%), 213 pathway
All unigenes matching Nr, UniProtKB, COG, KEGG	17,768 (56.8%)
Gene annotation against InterPro	25,457 (81.38%), 4,562 domains/families
Gene annotation against Pfam	12,909 (41.27%), 3,396 domains/families
Predicted coding sequence (CDS)	20,896 (66.8%)
All annotated unigenes	26,110 (83.47%)
Unigenes matching all seven databases	5,509 (17.61%)
GO annotation for Nr protein hits	4,706 (15.04%), 2,360 GO terms, 48 sub-categories
Biological process	2,315 (1,578 GO terms), 27 sub-categories
Cellular component	3,354 (270 GO terms), 10 sub-categories
Molecular function	2,809 (512 GO terms), 11 sub-categories

*Tm, T. multiceps*.

### Annotation Against Public Databases

In order to obtain and validate sequence-based annotations for all assembled unigenes, we employed Blastx for a sequence similarity search against the Nr database (animal protein of Nr and Drosophila protein of Nr databases), in addition to the UniProtKB/Swiss-Prot, UniProtKB/TrEMBL, COG, and KEGG databases, with an e-value threshold of 1.0^−5^. The results indicated that out of 31,282 unigenes, a total of 17,618 (56.32%) unigenes were annotated against animal protein of Nr, 5,925 (18.94%) against Drosophila protein of Nr, 14,350 (45.87%) obtained annotations against UniProtKB/Swiss-Prot, 16,286 (52.06%) against UniProtKB/TrEMBL, 6,653 (21.3%) received annotation against COG, and 11,645 (37.3%) against the KEGG database (Table 1). Altogether, BLAST searches against Nr, UniProtKB/Swiss-Prot, UniProtKB/TrEMBL, COG and KEGG databases identified 17,768 (56.80% of 31,282 unigenes) non-redundant unigenes of *T. multiceps*.

When the unigenes were first searched against the animal proteins of the Nr database [44], [45], the e-value distribution of the top hits in the animal proteins indicated that 36% of the mapped sequences had a significant similarity with a stringent threshold of less than 1.0^−50^, while 64% of the similar sequences ranged from 1.0^−5^ to 1.0^−50^ (Figure S1A). The similarity distribution revealed that 20% of the sequences had a similarity higher than 60%, whereas 80% of the hits had a similarity between 18% and 60% (Figure S1B). The species distribution showed that 48.73% of the unigenes had top matches and first hit against the sequences of *Schistosoma mansoni*, followed by *Schistosoma japonicum* (16.73%) and *Danio rerio* (2.09%), respectively (Figure S1C).

After being annotated against the COG database, 6,653 unigenes were classified into 25 functional categories and 1,221 COG terms. Among them, the cluster of ‘General function prediction only’ stood for the largest category (2,073, 17.43%), followed by 'Replication, recombination and repair’ (1,237, 10.40%) and ‘Transcription’ (1,087, 9.14%). In contrast, the clusters for ‘Nuclear structure’ (2, 0.017%), ‘Extracellular structures’ (3, 0.025%) and ‘RNA processing and modification’ (88, 0.74%) were the smallest categories (Figure S2).

**Figure 2:**
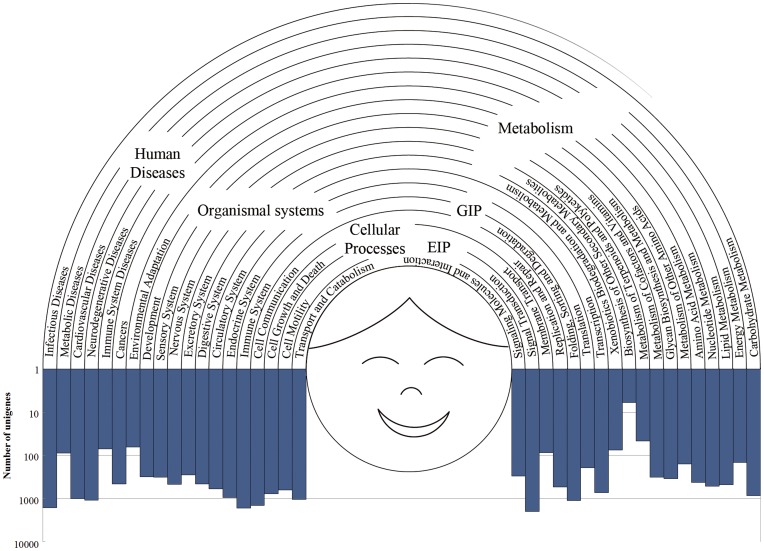
KEGG categories of *T. multiceps* unigenes. Overall, 11,645 unigenes were annotated against KEGG database. The GIP category represents ‘genetic information processing’ and EIP denotes ‘environmental information processing’.

The potential involvement in biological pathways of *T. multiceps* sequences was revealed by mapping against known proteins (equal to enzyme commission/EC number) of the KEGG database. Out of 31,282 assembled unigenes, a total of 11,645 homologous sequences were grouped into six categories, including ‘Metabolism’, ‘Genetic Information Processing’ (GIP), ‘Environmental Information Processing’ (EIP), ‘Cellular Processes’, ‘Organismal Systems’ and ‘Human Disease’ (Figure 2), and were assigned into 3,618 KEGG Orthologs (KO) terms and 213 KEGG pathways. Interestingly, the sub-category ‘Signal transduction’ (1,974, 16.95%) represented the majority of the ‘EIP’ category, followed by ‘Cancer’ (1,744, 14.88%) representing the majority of the ‘Human Disease’ category, and ‘Immune system’ (1,642, 14.10%) which represented the majority of the ‘Organismal System’ category (Figure 2, Table S1). Among the 213 pathways, the most abundant terms were: ‘MAPK signaling pathway’ (ko04010, 409), ‘Huntington's disease’ (ko05016, 413), ‘Pathway in Cancer’ (ko05200, 459), ‘Endocytosis’ (ko04144, 475), ‘Spliceosome’ (ko03040, 538) and ‘Regulation of actin cytoskeleton’ (ko04810, 630).

**Table 2:** The 30 most abundant InterPro domains/families in *T. multiceps* unigenes.

InterPro entry	InterPro domains/families	No. of *Tm* unigenes
IPR002110	Ankyrin repeat	226
IPR001680	WD40 repeat	142
IPR019781	WD40 repeat, subgroup	110
IPR001452	Src homology-3 domain	103
IPR003961	Fibronectin, type III	91
IPR001650	Helicase, C-terminal	86
IPR000504	RNA recognition motif domain	81
IPR007087	Zinc finger, C2H2-type	79
IPR019734	Tetratricopeptide repeat	79
IPR019782	WD40 repeat 2	75
IPR015880	Zinc finger, C2H2-like	74
IPR020683	Ankyrin repeat-containing domain	72
IPR000980	SH2 motif	60
IPR001715	Calponin homology domain	60
IPR013783	Immunoglobulin-like fold	57
IPR011009	Protein kinase-like domain	55
IPR000242	Protein-tyrosine phosphatase, receptor/non-receptor type	53
IPR003591	Leucine-rich repeat, typical subtype	53
IPR001478	PDZ/DHR/GLGF	51
IPR013032	EGF-like region, conserved site	49
IPR015943	WD40/YVTN repeat-like-containing domain	48
IPR002453	Beta tubulin	48
IPR001781	Zinc finger, LIM-type	47
IPR000719	Protein kinase, catalytic domain	46
IPR013083	Zinc finger, RING/FYVE/PHD-type	45
IPR002126	Cadherin	44
IPR017868	Filamin/ABP280 repeat-like	43
IPR000217	Tubulin	42
IPR011011	Zinc finger, FYVE/PHD-type	39
IPR006210	Epidermal growth factor-like	39

To identify domain-based annotations, unigenes were used to search the domain/families according to the InterPro and Pfam databases (e-value <1.0^−5^). As a result, 25,457 (81.38%) sequences obtained the entry description against InterPro and were categorized into 4,562 domains/families. Most domains/families were found to contain more than one unigene. According to the frequency of occurrence of *T. multiceps* unigenes contained in each InterPro domain, InterPro domains/families were ranked and the 30 most abundant InterPro domains/families are shown in Table 2. Among these InterPro domains/families, ‘Ankyrin repeat’ (226), ‘WD40 repeat’ (142) and its subgroup ‘WD40 repeat, subgroup’ (110), and ‘Srchomology-3 domain’ (103) were ranked as the most common domains/families. The majority of these were ‘Fibronectin, type III’ (91), the ‘Helicase, C-terminal’ (86), and the ‘RNA recognition motif domain’ (81). Moreover, 12,909 (41.27%) sequences could be mapped to entries in the Pfam database, defined by 3,396 different domains/families (Table 1). The five most abundant Pfam domains were ‘Protein kinase’ (327), ‘WD40’ (205), ‘RNA recognition motif_1’ domain (197), ‘HSP 70’ (99) and ‘Ank_2’ (88). The former three Pfam domains were included in the InterPro domain list mentioned above, and the 30 most abundant domains are shown in Table S2.

In total, 26,110 (83.47%) unigenes showed significant similarities to know proteins in the seven public databases (Table 1). After alignment against these databases, a total of 20,986 (66.8%) CDSs were inferred, 17,724 CDSs were predicted by Blastx and 3,172 CDSs by ESTScan.

### GO Functional Classification and GO Terms Comparison among Taeniidae Cestodes

**Figure 3:**
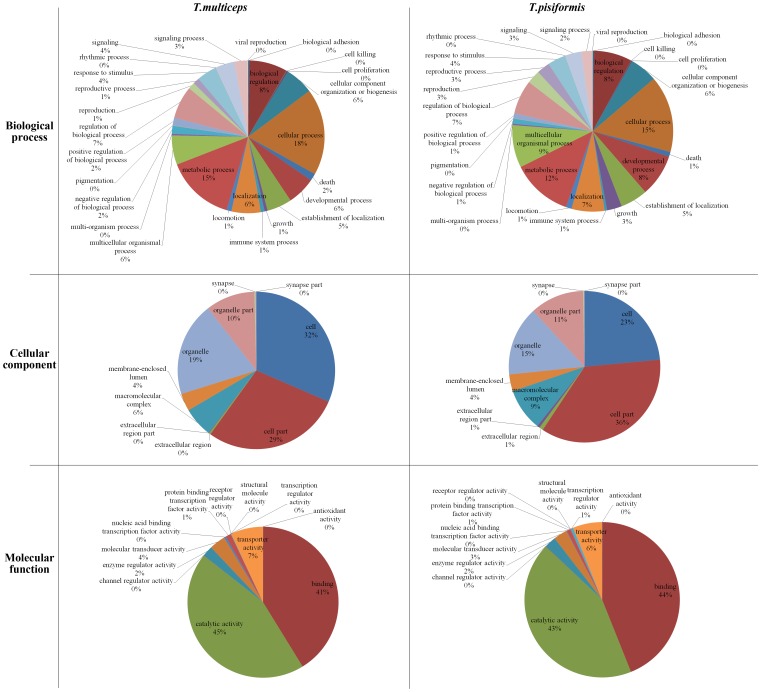
Transcriptomic Gene Ontology (GO) term comparison of *T. multiceps* and *T. pisiformis*. Pie chart illustrating similarities and differences between GO terms (according to the categories ‘cellular component’ and ‘molecular function’ and ‘biological process’) assigned to peptides from *T. multiceps* and *T. pisiformis* inferred from transcriptomic data.

Of the 17,618 Nr hits, a total of 4,706 sequences were assigned to 2,360 non-redundant GO terms according to the Blast2GO program. All GO terms were allocated into three main GO categories (including three categories: biological process, cellular component and molecular function) and 48 sub-categories (Table 1 and Figure 3, left). Biological process made up the majority (1,578 GO terms, 2,315 unigenes) followed by molecular function (512 GO terms, 2,809) and cellular component (270 GO terms, 3,354). Among the 10 sub-categories of cellular components, GO terms were predominantly associated with ‘cell’ (3,324, 31.51%), ‘cell part’ (2,999, 28.43%) and ‘organelle’ (2,036, 19.3%) (Figure 3, left). Furthermore, a comparative analysis of GO terms based on the transcriptome unigenes of *T. multiceps* and *T. pisiformis* is shown in Figure 3 [37]. The major parts of each category in GO classification between *T. multiceps* and *T. pisiformis* showed the same sub-categories and a similar percentage, but still revealed their qualitative and quantitative differences [34].

**Figure 4:**
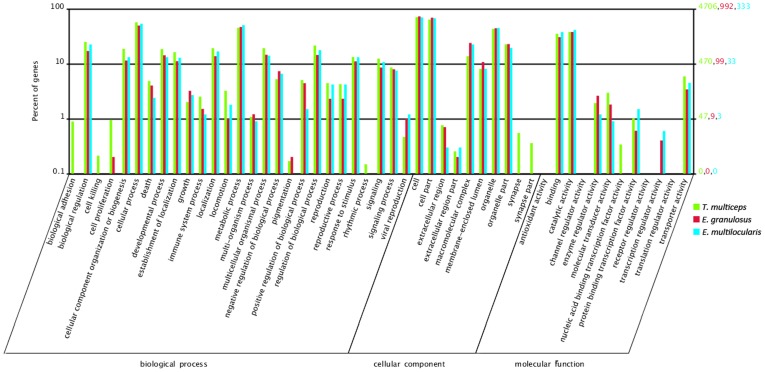
GO terms similarity distribution among *T. multiceps*, *E. granulosus* and *E. multilocularis*. Bar graph plotted using a web-based tool, WEGO.

To enabled a further analysis of the comparative GO classification with other cestodes, we searched the ESTs from the two homologous species, *E. granulosus* and *E. multilocularis* (genus Echinococcus, family Taeniidae), on NCBI. The 9,701 ESTs of *E. granulosus* received 5,876 GO annotations, whereas 1,168 ESTs of *E. multilocularis* obtained 1,979 GO annotations against Gene Ontology. WEGO showed the similar percentages of GO terms among these three cestode species (Figure 4).

### Common Genes Found Among Selected Zoonotic Intestinal Parasites

**Figure 5:**
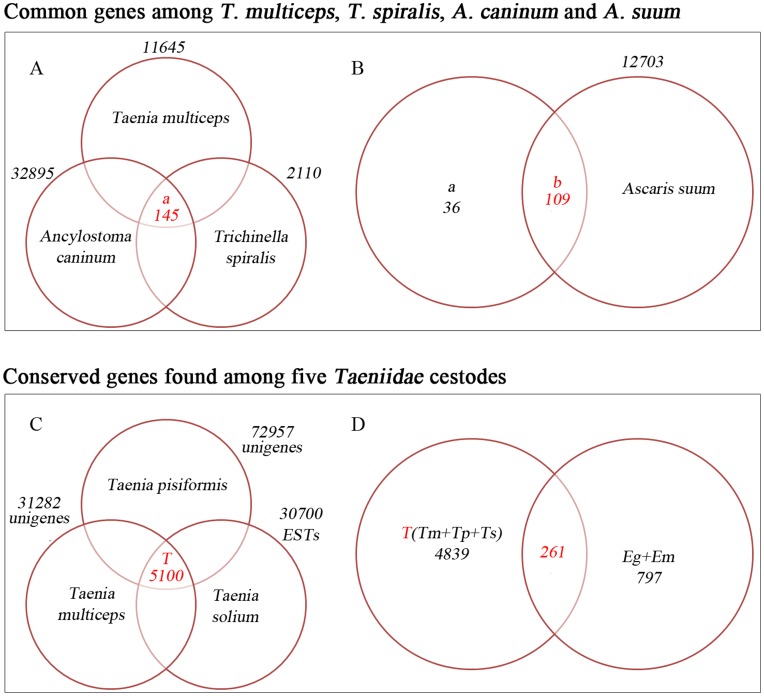
Venn diagram showing the overlap sequences among four intestinal parasites and five Taeniidae cestodes. (A) 145 common genes shared by *T. multiceps*, *T. spiralis* and *A. caninum*. (B) 109 common genes shared by *T. multiceps*, *T. spiralis*, *A. caninum* and *A. suum*. (C) 5,100 common genes among *T. multiceps*, *T. pisiformis* and *T. solium*. (D) 261 conserved genes between *T. multiceps*, *T. pisiformis*, *T. solium*, *E. granulosus* and *E. multilocularis*.

To find essential genes and understand the biology of intestinal helminths further, we compared four selected zoonotic intestinal parasites (*Taenia multiceps*, *Ancylostoma caninum*, *Trichinella spiralis* and *Ascaris suum*). The available transcript datasets of four species of zoonotic intestinal parasites were chosen to detect common genes that may be involved in adapting to intestinal parasitic life based on KEGG annotations. Of the 32,895 annotated nucleotide ESTs (40,66% of 80,905) of *A. caninum*, 11,645 annotated unigenes (37.26% of 31,282) of *T. multiceps* and 2,110 annotated CDSs (12.88% of 16,380) of *T. spiralis*, 145 common genes were found from the overlapping annotated part (identity ≥80%) of *A. caninum*, *T. spiralis* and *T. multiceps* (Figure 5A). Together with 12,703 annotated contigs (19.26% of 65,952) of A. suum, 109 common genes were found from overlapping parts (Figure 5B) of the four species of intestinal parasites, which could constitute essential genes or potential drug targets. The 109 common genes IDs with corresponding annotations among *T. multiceps*, *T. spiralis*, *A. caninum* and *A. suum* are shown in Table S3. Here, one (unigene 18109) of the common genes was identified using RT-PCR, with the accession number GU205474 in GenBank.

### Conserved Genes between *Taenia* and *Echinococcus* Tapeworms

In order to obtain a more detailed understanding of Taeniidae cestode biology and reveal potential drug target genes, we compared five important cestodes by making use of currently available datasets. After comparing three cestodes of the Taenia genus (31,282 unigenes of *T. multiceps*, 72,957 unigenes of *T. pisiformis* and 30,700 ESTs of *T. solium*), we obtained 5,100 common genes (Figure 5C). Of these, 3,000 were annotated by the KEGG database (common genes are shown in Table S4). When the 5,100 Taenia common genes were combined with the 1,058 ESTs of Eg+Em (conserved ESTs between *E. granulosus* and *E. multilocularis*) [30], 261 conserved genes were detected (Figure 5D), and 204 obtained KO annotations (Table S5). Some of the common genes were involved in *T. multiceps* survival and parasite-host interactions, supporting the development of drug targets/vaccines and phylogenetic relationship analyses between *Echinococcus* and *Taenia* tapeworms. In addition, we validated one promising drug target (unigene 1299) using RT-PCR to support the further analysis of its protein structure and function (GenBank accession number: GU205473).

## Discussion

In this study, a reliable and substantial transcriptome dataset of the adult stage of *T. multiceps* was produced by Illumina sequencing and Trinity assembling. The percentage of both contigs (≥500 bp) and unigenes (≥500 bp) was higher than 60%, and the gap rate of all contigs and unigenes was as low as 0%. Unlike previous studies, our results showed that the mean length (920 bp) of *T. multiceps* unigenes was shorter than the contigs (974 bp), which might be due to the higher frequency of long sequences in contigs than in unigenes, whereas the number of contigs in each length extent was higher than that of unigenes. The reason for this is that one unigene has multiple transcripts, due to alternative splicing in eukaryotes. Of 31,282 unigenes, 26,110 (83.47%) could be annotated by seven public databases, whereas the remaining 16.53% unaligned unigenes are most likely contain Taenia- or cestode-specific genes [46]. Compared with our previous study on *T. pisiformis* [30], in which 35.23% of all distinct sequences assembled by de novo SOAP had an annotation against the Nr database, the higher percentage of 56.32% found in this study might be partially due to the higher percentage of long sequences distributed in our unigenes (mean length of 920 bp versus a mean length of 398 bp in *T. pisiformis*). This is in accordance with a previous report that showed longer contigs are more likely to obtain BLAST matches in protein databases [47]. Moreover, a total of 20,896 CDS for *T. multiceps* was predicted from this transcriptome dataset, with an approximate 200-fold coverage of the available proteins (for this stage/species) in the public databases. All of these results demonstrate the high quality and effectiveness of Illumina paired-end sequencing technology and the Trinity assembler.

Throughout the biological pathway sub-categories, ‘signal transduction’ (1,974 unigenes) of the environmental information processing (EIP) category was the most highly represented in the transcriptome of *T. multiceps* (6.3%). In this sub-category, the ‘Wnt signaling pathway’ was one of the most abundant pathways and was particularly interesting. A comparative analysis of Wnt signaling components in parasitic and free-living flatworms (including *H. microstoma*, *E. multilocularis*, *E. granulosus*, *S. mansoni* and *Schmidtea mediterranea*) has been conducted and a hypothesis of Wnt gene loss in flatworms has been proposed [48]. It was found that Wnt1 was expressed only in adults of *H. microstoma*, whereas Wnt2 was expressed only in larvae. Wnt1 was known to play a role as a segment polarity gene in adult worms [48]. Surprisingly, neither Wnt1 nor Wnt2 were found in this adult *T. multiceps* transcriptome annotation. This may have been due to the limitation of Wnt annotations in the currently available databases, or may reveal that the presence of Wnt1 and Wnt2 differ between species. Further research is necessary to validate the role of Wnt genes in the development of *T. multiceps* and their loss in cestodes.

After the transcript comparison of four species of zoonotic intestinal helminths, we narrowed the scope of the gene group to find genes that might be used for adapting to intestinal parasitic life. The 109 genes in the common part were still imperfect due to the limited available dataset. However, they did contain conserved genes, essential genes for parasite survival or genes related to parasite-host interactions. The majority of these common genes were α-tubulin and actin (see Table S3 for the corresponding unigene number), which were conserved structural proteins and were essential in parasites.

Interestingly, we found that phosphoenolpyruvate carboxykinase (PEPCK: unigene 17356) might be involved in parasite-host interaction. PEPCK is a key enzyme in malic acid disproportionation, which is the principle process for the continuous metabolism of phosphoenolpyruvate (PEP). PEP is generated from anaerobic glycolysis, in order that intestinal parasitic helminths can obtain ATP in anaerobic conditions [49]. Therefore, the lack of this important enzyme would interrupt parasite glycometabolism. As PEPCK has significant differences in the molecular properties between host and parasites, the parasite PEPCK molecular characteristics need further identification as a possible drug target [50]. The 3D structure of *A. suum* PEPCK was predicted by Verma et al. [50], but further analysis of PEPCK in intestinal helminths will be necessary to investigate whether a parasite-specific drug that is minimally toxic to the host can be found.

*T. multiceps*, *T. solium*, *E. granulosus, E. multilocularis* and *T. pisiformis* are five important Taeniidae cestodes, of which the former four species are zoonotic parasites that cause huge economic losses and threaten human health [12], [29]. The currently available sequences for *T. multiceps* are poor and less than 10 candidate antigens exist as drug targets, including Tm18 [EF672035], Tm16 [EF672037], 45 m [FJ461729], TPx [HQ888859] and Tm7 [FJ603044]). In this study, we used relatively large datasets that contained 31,282 unigenes of *T. multiceps* and 72,597 unigenes of *T. pisiformis* obtained by high-throughput sequencing, and 30,700 ESTs of *T. solium* obtained by cDNA library construction, to bring to light the scope of potential drug targets/candidate vaccines. Conserved *Taenia* genes and essential genes for *Taenia* survival were contained by this scope of common genes. There might be other new *Taenia*-specific genes in the remaining 2,100 un-annotated common genes.

Together with the Eg+Em common ESTs, 261 conserved genes were obtained between *Taenia* and *Echinococcus* tapeworms. Among 204 annotated conserved genes, we also found PEPCK (unigene 17356). As the five Taeniidae cestodes are also intestinal parasites, the PEPCK might play the same role as discussed above and could be a promising drug target if it is minimally toxic to the host. Furthermore, PEPCK has been considered as a new marker of the phylogenetic relationship within *Echinococcus* and *Taenia* tapeworms [51], and this current finding of PEPCK in *T. multiceps* will help in the study of cestode phylogenetic relationships.

The 261 conserved genes were believed to contain necessary genes for the five Taeniidae cestodes that could help drug target/vaccine finding. Unigene 1299 (see Table S5) of these conserved genes was particularly important and valuable. Unigene 1299 was annotated as FABP3. FABPs functions to enable these five cestodes to obtain long-chain fatty acids and cholesterol from the host to substitute for their lack of de novo synthesis of most lipids [52]. Two further reasons support the use of FABP as a promising candidate vaccine: 1) all five Taeniidae cestodes, which live in the intestinal tract, simulate the host intestinal mucosal immunity to secrete IgA; and 2) FABP of *E. granulosus* can induce the host to produce IgA, IgG1 and IgG2a [53]. As a result of the potential of this unigene, we amplified the full-length CDS of *T. multiceps* FABP3 using RT-PCR.

In this in-depth study, we obtained a broad transcriptome dataset of the adult stage of *T. multiceps* using Illumina paired-end sequencing technology and a Trinity de novo assembler without a reference genome. A total of 31,282 unigenes was produced with 26,110 sequences having annotations against seven public databases. We have demonstrated the feasibility and advantage of using a Trinity assembler. The common genes found among four zoonotic intestinal parasites (*T. multiceps*, *T. spiralis*, *A. caninum* and *A. suum*) and the comparative transcript analysis with *T. pisiformis*, *T. solium*, *E. granulosus* and *E. multilocularis* established a substantial platform for the better understanding of *T. multiceps* survival and development, further study of parasite-host interactions, and the development of future drug targets/vaccines and phylogenetic relationship analyses within *Echinococcus* and *Taenia* tapeworms.

## Materials and Methods

### Sample Preparation

The larvae (coenurus) (kindly provided by Yingdong Yang, Panzhihua, China) were collected from the brain of a naturally infected goat at an organic farm from Sichuan, China. The infection experiment was followed by larval morphological identification, and was performed by administering 20 larvae of *T. multiceps* into two parasite-free beagle dogs. Once the gravid proglottid expelled in the feces of infected dogs appeared on day 28 post-infestation, adult *T. multiceps* were immediately removed from the small intestine and washed thoroughly in physiological saline solution (37°C) to avoid host contamination; they were then transferred into liquid nitrogen and stored at −80°C until further use. All animals from which specimens were collected were handled in accordance with the animal protection law of the People's Republic of China (a draft of an animal protection law in China was released on September 18^th^, 2009). This study was approved by the National Institute of Animal Health Animal Care and Use Committee at Sichuan Agricultural University (approval number 2010–018).

### RNA Isolation and Illumina Sequencing

A paired-end transcriptome sequencing (RNA-Seq) [22] was employed. Total RNA was isolated from adult *T. multiceps* (n = 6) using Trizol (Invitrogen, Carlsbad, CA), following the manufacturer's instructions. The integrity of total RNA was verified using Agilent 2100 with the RNA integrity number (RIN). Polyadenylated (polyA) RNA was purified from 40 µl of total RNA using Sera-Mag oligo (dT) beads, fragmented into small pieces by fragmentation buffer, reverse-transcribed using random hexamers and reverse transcriptase, and then end-repaired with adapter primer attached and adaptor-ligated by the addition of a specific adapter, according to the manufacturer’s protocol (Illumina). These ligated products were purified and amplified with PCR to create the final cDNA library [45]. The cDNA library was sequenced by Beijing Genomics Institute (BGI)-Shenzhen, Shenzhen, China, on a HiSeq™ 2000 (Illumina), according to manufacturer’s instructions. The transcriptome raw reads dataset has been submitted to the NCBI Short Read Archive (http://www.ncbi.nlm.nih.gov/Traces/sra_sub/sub.cgi) with the accession number: SRA048944.

### Bioinformatic Analysis

A new Trinity de novo transcriptome assembler [26] was selected to assemble the sequence data from Illumina sequencing for *T. multiceps*. Reads that have a certain length overlap area were joined (the best method of joining was chosen by Trinity) into longer fragments, which are called contigs without gaps. TIGR Gene Indices clustering tools (TGICL) [54] were used to splice sequences and remove redundant sequences, and then unigenes without gaps could be obtained until they could not be further elongated. Reads per kb per million reads (RPKM) [55] were used to show the expression quantity, thus avoiding the influence of sequencing length and differences. The assembled unigenes (longer than 300 bp) are available from the Transcriptome Shotgun Assembly Sequence Database (TSA) at the NCBI with the following accession numbers: JR916739 -JR948020.

Unigene sequences were first aligned to the protein databases Non-redundant (Nr), UniProtKB/Swiss-Prot, UniProtKB/TrEMBL, Cluster of Orthologous Groups (COG) and the Kyoto Encyclopedia of Genes and Genomes (KEGG) databases by Blastx, using an e-value threshold of 1.0^−5^. InterProScan (http://www.ebi.ac.uk/InterProScan/) [56] and HMMER (http://hmmer.janelia.org/) were used to obtain domain-based annotation by InterPro (http://www.ebi.ac.uk/interpro/) [57] and Pfam version 25.0 (March 2011, 12,273 families) (http://Pfam.sanger.ac.uk) [58] terms, as previously described (Shi et al. 2011). The unigenes were tentatively annotated according to the known sequences with the highest sequence similarity. The annotated unigenes direction and CDSs were identified by the best alignment results. ESTScan [59] was used to predict the coding sequences (CDS) and the sequence direction when unigenes were unaligned to any of the databases.

Of the Nr annotations, Gene Ontology (GO) annotations of unigenes were obtained using Blast2GO software (version 2.3.5, http://www.blast2go.de/) [60] (e-value <1.0^−5^) and were assigned into three ontologies (molecular function, cellular component, biological process) (http://www.geneontology.org/). WEGO software (http://wego.genomics.org.cn/cgi-bin/wego/index.pl) [61] was used to perform GO functional classification for all unigenes and to display the distribution of gene functions and the similarity and difference among *T. multiceps*, *E. granulosus* and *E. multilocularis* on a macro level.

### Comparative Transcripts Analysis

To compare GO classification among Taeniidae cestodes, the unigenes of *T. multiceps* (31,282) and *T. pisiformis* (72,957) were provided by our laboratory. *T. pisiformis* unigenes were also produced by the BGI center (see Dataset S1 in the previous study [30]), while 9,701 ESTs of *E. granulosus* and 1,168 ESTs of *E. multilocularis* were downloaded from http://www.ncbi.nlm.nih.gov/Taxonomy/Browser. We mapped these unigenes and ESTs to Gene Ontology (e-value <1.0^−5^). For four intestinal parasites common genes finding, 80,905 nucleotide ESTs of *A. caninum* were downloaded from http://www.ncbi.nlm.nih.gov/Taxonomy/Browser/wwwtax.cgi?id=29170, 16,380 CDSs in 9,267 contigs of *T. spiralis* [GenBank contigs: ABIR02000001–ABIR02009267; GenBank proteins: EFV46182–EFV62561] [62] and 62,592 contigs of *A. suum* were downloaded from ‘Ascaris_cDNA_All_v1.fa.gz - *Ascaris suum* cDNA assembly’ (http://www.nematode.net/NN3_frontpage.cgi?navbar_selection=home&subnav_selection=asuum_ftp). All transcripts were chosen to be mapped to the known proteins in KEGG (e-value <1.0^−5^) and the further comparative analysis among the four different species of intestinal parasites were based on KEGG annotations. The overlap for common sequences was obtained with an identity threshold of ≥80%.

### Common Genes Found between *Taenia* and *Echinococcus*

Based on the previous study of the *T. pisifomis* transcriptome, we choose 31,282 and 72,597 unigenes from *T. multiceps* and *T. pisiformis* [30], and 30,700 ESTs available from *T. solium* [29] to find the common genes among *Taenia* spp. When the common *Taenia* genes were combined with 1,058 ESTs from Eg+Em (see previous study [30]), we obtained the common genes shared between *Taenia* and *Echinococcus*. Finally, the common genes found among *Taenia* (*T. multiceps*, *T. pisiformis* and *T. solium*) and conserved genes between *Taenia* and *Echinococcus* (*E. granulosus* and *E. multilocularis*) were aligned to the KEGG database using the Blastx algorithm.

### Validation of the Transcriptome CDS of *T. multiceps*

The full-length CDS of two potential unigenes were amplified by RT-PCR using cDNA of adult *T. multiceps* (primers and annealing temperature are shown in Table S6). Primers of the two unigenes were designed using Primer premier 5.0.

## Supporting Information

Figure S1 **Characteristics of homology search of all assembled unigenes of T. multiceps against the Nr database.** (A) e-value distribution. (B) Similarity distribution of the top BLAST hits for each unigene. (C) Species distribution is shown as the percentage of the total homologous unigenes with a threshold e-value of 1.0^−5^.
(TIF)

Figure S2 **COG function classification of the** ***T. multiceps*** **sequences.**
(TIF)

Table S1 **Detailed KEGG pathway categories of the** ***T. multiceps*** **unigenes.**
(XLS)

Table S2 **The 30 most abundant Pfam domains/families in** ***T. multiceps*** **unigenes.**
(XLS)

Table S3 **The 109 common genes among** ***T. multiceps*** **,** ***T. spiralis*** **,** ***A. caninum*** **and** ***A. suum*** **.**
(XLS)

Table S4 **5,100 common genes among** ***T. multiceps*** **,** ***T. pisiformis*** **and** ***T. solium*** **.**
(RAR)

Table S5 **261 conserved genes among** ***T. multiceps*** **,** ***T. pisiformis*** **,** ***T. solium*** **,** ***E. granulosus*** **and** ***E. multilocularis*** **.**
(RAR)

Table S6 **Primers were designed for RT-PCR of two unigenes from the adult** ***T. multiceps*** **transcriptome.**
(DOC)
