# Supplementary figures and images for: Detailed Transcriptome Description of the Neglected Cestode Taenia multiceps
Source: PLoS One. 2012 Sep 25;7(9):e45830. doi: 10.1371/journal.pone.0045830 (PMC3458062; doi:10.1371/journal.pone.0045830)

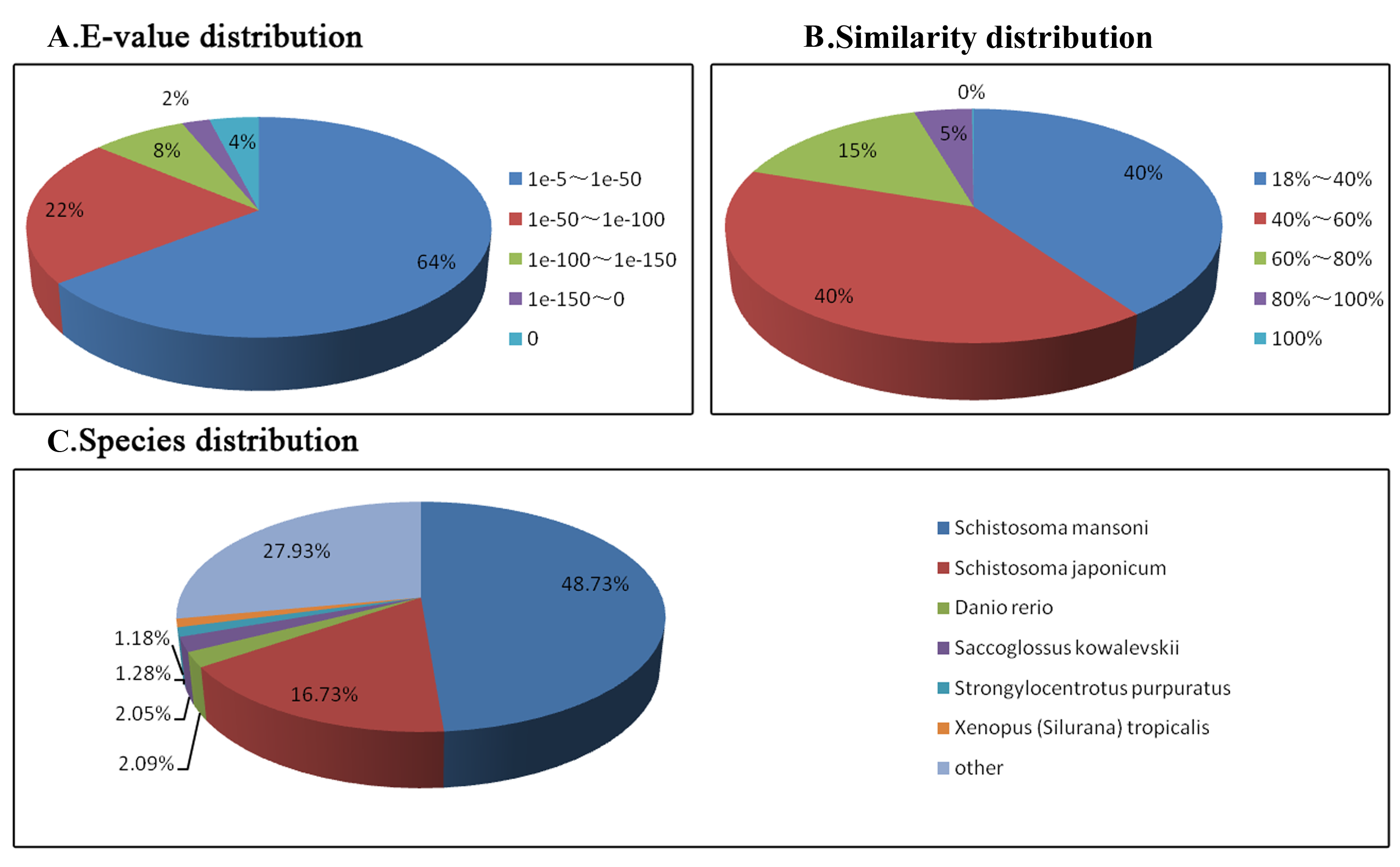

Supplement: Figure S1 — Characteristics of homology search of all assembled unigenes of T. multiceps against the Nr database. (A) e-value distribution. (B) Similarity distribution of the top BLAST hits for each unigene. (C) Species distribution is shown as the percentage of the total homologous unigenes with a threshold e-value of 1.0−5. (TIF) [file pone.0045830.s001.tif]

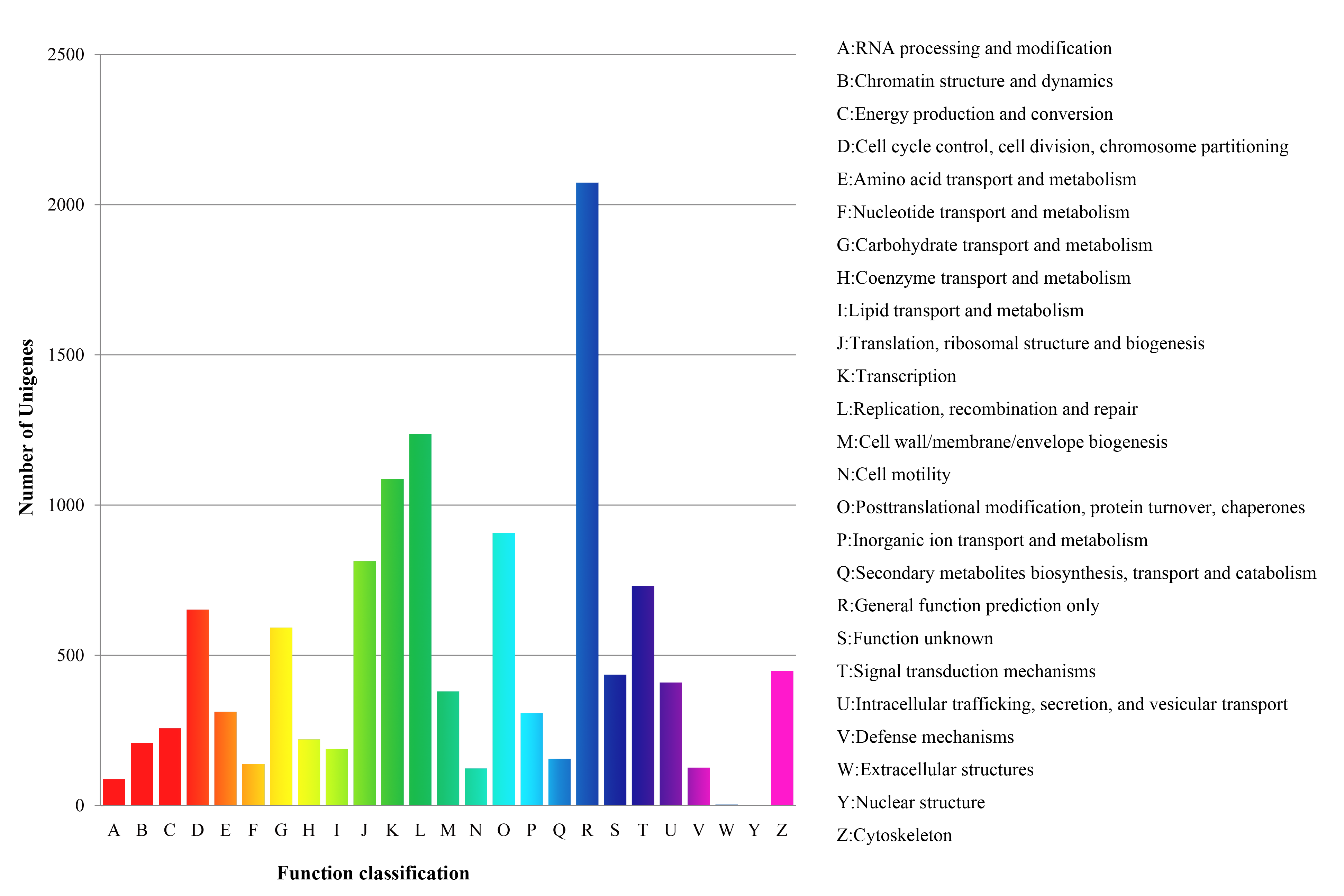

Supplement: Figure S2 — COG function classification of the T. multiceps sequences. (TIF) [file pone.0045830.s002.tif]
