# Supplementary material for: Detailed Transcriptome Description of the Neglected Cestode Taenia multiceps
Source: PLoS One. 2012 Sep 25;7(9):e45830. doi: 10.1371/journal.pone.0045830 (PMC3458062; doi:10.1371/journal.pone.0045830)
Supplement: Table S6 — Primers were designed for RT-PCR of two unigenes from the adult T. multiceps transcriptome. (DOC) [file pone.0045830.s008.doc]

**Table S6.** Primers were designed for RT-PCR of two unigenes from adult *Taenia multiceps* transcriptome.

|  | Primer name | | | Primer sequence (5’-3’) Annealing temperature |
| --- | --- | --- | --- | --- |
| Fabp3 | Forward | | ATGGAGCCATTCATCGGT 56℃ | |
|  | Reverse | | TCCCTTACGCTGCCTTA | |
| HSP70 | Forward | | ATGTCGAAAGGACCAGC 52℃ | |
|  | Reverse | CTGACTCTGACTGTCGTTG | | |
